# Hepatopulmonary Fistula Secondary to an Amebic Liver Abscess: A Case Report

**DOI:** 10.7759/cureus.107912

**Published:** 2026-04-28

**Authors:** Lina M Charry Jiménez, Johnier Quintero Ropero, Andrés F Vargas Camacho, Jose R Prada Davila

**Affiliations:** 1 Department of Internal Medicine, Fundación Oftalmológica de Santander (Clínica FOSCAL), Universidad Autónoma de Bucaramanga, Bucaramanga, COL

**Keywords:** ala, amebiasis, amebic liver abscess, hepatopulmonary fistula, liver abscess

## Abstract

Hepatopulmonary fistula (HPF) is an uncommon complication of amebic liver abscess (ALA) and is associated with high morbidity and mortality when not promptly recognized. We report a case of a 39-year-old man with no relevant medical history who initially presented with diarrhea, followed by fever, right upper quadrant pain, and cough with brownish sputum. Contrast-enhanced computed tomography (CT) revealed a large liver abscess with diaphragmatic disruption and secondary pulmonary extension. The patient was managed with video-assisted thoracoscopic surgery (VATS), including pulmonary decortication, wedge resection, diaphragmatic plication, and subdiaphragmatic drainage, in addition to targeted antibiotic therapy, achieving favorable clinical outcomes. This case underscores the importance of suspecting HPF in patients with ALA who develop atypical respiratory symptoms, highlights the value of CT as a key diagnostic tool, and emphasizes the role of a multidisciplinary approach. From an endemic setting, this report contributes valuable evidence to the international literature regarding a rare but potentially life-threatening complication.

## Introduction

Amebiasis, caused by *Entamoeba histolytica*, remains a significant health problem in tropical regions, with a high prevalence of amebic liver abscesses (ALAs), which represent the most common extraintestinal manifestation of this disease. Although most ALAs follow an uncomplicated course, uncommon presentations have been described, including rupture into adjacent abdominothoracic structures, resulting in biliary, vascular, or hepatic fistulas (hepatobronchial, hepatogastric, hepatoenteric, and hepatocolonic fistulas) or extension into the pleuropulmonary space [1].

Among these complications, hepatopulmonary fistula (HPF) is an exceptionally rare entity [2], defined as an abnormal communication between the hepatic parenchyma and the bronchial tree or pleura, often traversing the diaphragm [3,4]. Although its true incidence remains unclear, only a limited number of cases have been reported in the literature, most commonly as isolated case reports or small case series [5,6]. 

This case is particularly relevant in emphasizing the need to maintain a high index of clinical suspicion, as this entity may mimic other pulmonary conditions and delay its diagnosis, especially in endemic regions; moreover, in selected cases, conservative or minimally invasive management may not be sufficient, highlighting its potential severity and the importance of timely multidisciplinary intervention.

The pathophysiology of HPF is attributed to progressive diaphragmatic erosion caused by mass effect and local inflammation from a subphrenic abscess, eventually leading to communication with the pleura and lung; alternatively, it may result from direct invasion of the pulmonary parenchyma from an adjacent hepatic focus [3,4]. Clinically, patients typically present with fever, right upper quadrant pain, cough, and purulent sputum; when the communication is bronchobiliary, bilioptysis constitutes the pathognomonic finding [4,7]. 

In Latin America, where amebiasis remains endemic, reporting uncommon complications has particular epidemiological and clinical relevance. Studies from the region have reported complication rates of up to 20% in patients with ALA, highlighting the importance of recognizing atypical and severe presentations [8]. In this context, we present the case of a patient with an ALA complicated by HPF, emphasizing the importance of timely clinical and imaging-based diagnosis and a multidisciplinary management approach to reduce the associated morbidity and mortality.

## Case presentation

A 39-year-old Colombian man, with no relevant medical history, presented with a one-month history of illness characterized by watery diarrhea, followed by persistent fever up to 39°C, right upper quadrant pain radiating to the ipsilateral costal and dorsal regions, and productive cough with brownish sputum. At primary care centers, the patient received empiric treatment with trimethoprim-sulfamethoxazole for presumed acute bacterial diarrheal illness without clinical improvement. 

The patient was subsequently hospitalized at a secondary-level facility with a presumptive diagnosis of pneumonia. Contrast-enhanced abdominal computed tomography (CT) demonstrated a hepatic collection measuring 12 × 11 × 10 cm, with an estimated volume of approximately 680 mL calculated using the ellipsoid formula based on the reported orthogonal diameters, with right subdiaphragmatic extension and involvement of the adjacent basal lung parenchyma. Antimicrobial therapy with ciprofloxacin and metronidazole was initiated, and the patient was referred to our institution for percutaneous drainage.

On admission, he was hemodynamically stable (blood pressure 125/83 mmHg, heart rate 63 beats/min, respiratory rate 20 breaths/min, oxygen saturation 95% on room air, temperature 36.4°C), alert, and afebrile, with hypoventilation and crackles at the right lung base. Laboratory evaluation revealed mild anemia without leukocytosis or neutrophilia, thrombocytosis, elevated C-reactive protein, normal renal function, elevated alkaline phosphatase and mild transaminase elevation, and prolonged partial thromboplastin time (Table 1). 

**Table 1:** Laboratory findings. These results show a pattern of hepatobiliary involvement with elevated liver enzymes and bilirubin levels, accompanied by a significant systemic inflammatory response.

Category	Parameter	Result	Reference Range	Interpretation
Renal function	Creatinine	0.92 mg/dL	0.5-0.95	Within normal limits
	Blood urea nitrogen (BUN)	6.3 mg/dL	8.0-23	Mildly decreased
Glucose metabolism	Blood glucose	98 mg/dL	70-100	Within normal limits
Inflammation	C-reactive protein (CRP)	34.93 mg/L	0.6-5.0	Markedly elevated, consistent with systemic inflammation
Liver function	Aspartate aminotransferase (AST)	85 U/L	<40	Elevated
	Alanine aminotransferase (ALT)	72 U/L	<41	Elevated
	Alkaline phosphatase (ALP)	320 U/L	40-130	Markedly elevated, suggestive of cholestatic involvement
	Total bilirubin	1.8 mg/dL	<1.2	Elevated
	Direct bilirubin	1.1 mg/dL	<0.3	Elevated
	Albumin	3.7 g/dL	3.5-5.0	Within normal limits
Complete blood count	Hemoglobin	12.7 g/dL	13-17	Mild anemia
	Leukocytes	8,820/mm³	4,000-11,000	Within normal limits
	Neutrophils	6,130/µL	2,000-7,000	Within normal limits
	Lymphocytes	1,250/µL	1,500-4,000	Mild lymphopenia
	Platelets	704,000/mm³	150,000-450,000	Marked thrombocytosis, likely reactive
Coagulation	Prothrombin time (PT)	12.2 s	9.7-13.5	Within normal limits
	Activated partial thromboplastin time (aPTT)	33.1 s	22.5-30.5	Mildly prolonged, without significant coagulopathy
	International normalized ratio (INR)	1.2	0.9-1.3	Within normal limits

**Figure 1:**
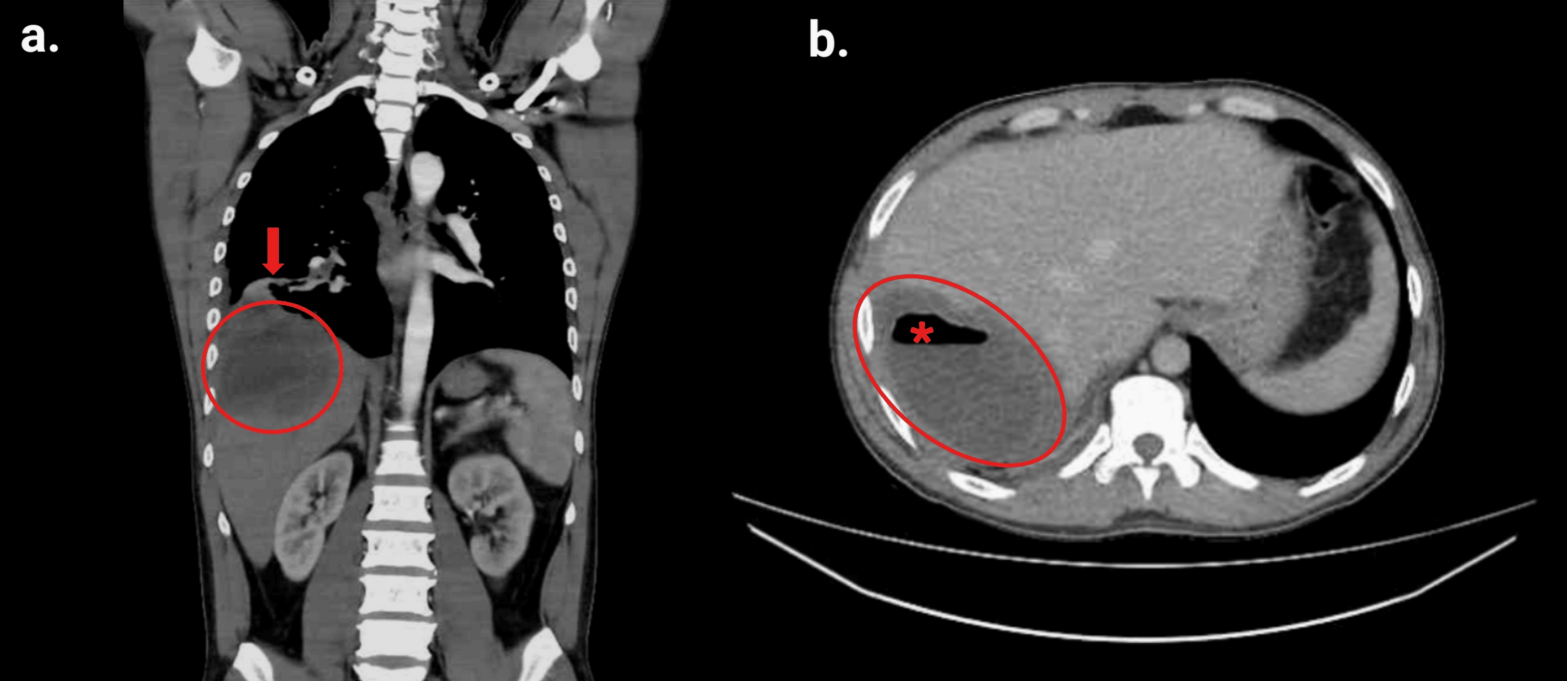
Contrast-enhanced abdominal and pelvic CT: (a) coronal view and (b) axial view. A multiloculated hypodense lesion with thick walls and an internal air-fluid level (red asterisk), consistent with a hepatic abscess (red circles), is identified in segments VII and VIII of the right hepatic lobe. The lesion measures 12.3 × 7.7 × 8.1 cm in the anteroposterior, transverse, and craniocaudal dimensions, respectively. Loss of the normal plane of separation between the hepatic parenchyma and the diaphragm is noted, with identification of a small posterior diaphragmatic defect (red arrow) measuring approximately 4.7 mm, associated with a cavitary lesion suggestive of a right basal pulmonary abscess (see Figure 2), raising suspicion of hepatopulmonary communication. CT: computed tomography

**Figure 2:**
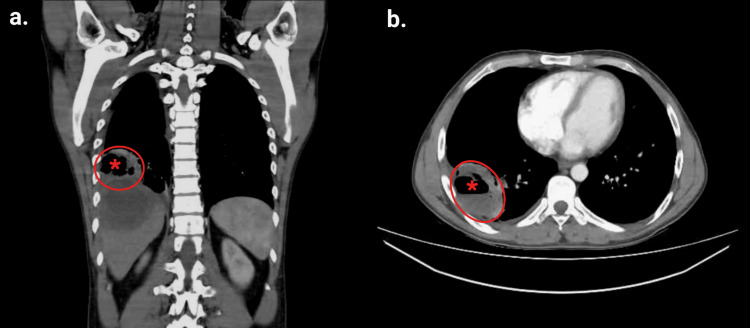
Contrast-enhanced chest CT: (a) coronal view and (b) axial view. A hypodense collection consistent with a pulmonary abscess (red circles) is observed in the anterior and lateral basal segments of the right lower lobe, with an internal air-fluid level (red asterisks) and irregular margins, suggestive of direct extension from the hepatic abscess with secondary infection. The lesion measures 5.1 × 7.8 × 7.3 cm in the anteroposterior, transverse, and craniocaudal dimensions, respectively, and is associated with pleural effusion and adjacent ground-glass opacity. CT: computed tomography

A repeat thoracoabdominal CT scan demonstrated a hepatic abscess with basal pulmonary extension through a defect in the posterior aspect of the ipsilateral diaphragm (Figures 1-2). 

Interventional radiology deferred percutaneous drainage due to the large size of the lesion, its subdiaphragmatic extension, and the suspected hepatopulmonary communication, which limited adequate source control and safe management of the thoracic component. Therefore, surgical management by the thoracic surgery service was indicated. Antimicrobial therapy was adjusted to intravenous levofloxacin 750 mg every 24 hours, while metronidazole was continued. Following the identification of *Giardia duodenalis* on stool examination, and given that metronidazole provides coverage against this pathogen, a single oral dose of albendazole 400 mg was administered as adjunctive antiparasitic therapy. 

Video-assisted thoracoscopic surgery (VATS) was performed, including pulmonary decortication, wedge resection of the right lower lobe to remove necrotic infected tissue, diaphragmatic plication to reinforce the diaphragmatic defect, subdiaphragmatic drainage for source control, and closed thoracostomy with Blake drain (Ethicon Inc., Somerville, USA) placement. Intraoperatively, an approximately 1 cm diaphragmatic defect was identified, establishing communication between the hepatic abscess and the thoracic cavity, along with a pulmonary abscess at the base of the right lower lobe. Blood cultures were obtained at admission, and intraoperative cultures from the hepatic abscess and pleural space were also collected. All cultures remained negative, likely reflecting prior antimicrobial exposure before microbiological sampling. 

In the immediate postoperative period, antimicrobial therapy with levofloxacin and metronidazole was maintained. Due to persistent fever and a suboptimal clinical response, antibiotic coverage was subsequently escalated to piperacillin/tazobactam 4.5 g intravenously every eight hours, given the high suspicion of secondary pyogenic bacterial infection. The diagnosis of ALA was established based on the combination of clinical presentation, radiological findings, and positive serologic testing for *Entamoeba histolytica* (Table 2). 

**Table 2:** Infectious disease workup. These findings support an amebic hepatobiliary infection, consistent with a non-pyogenic etiology. RBC: red blood cell

Category	Test	Result	Interpretation
Serology	*Entamoeba histolytica *antibodies	>1:1280	Positive (≥1:320)
Stool examination	Macroscopic	Brown color, soft consistency, pH 5	Within expected range
	Chemical	Reducing sugars (-), glucose (-), fecal occult blood (+)	Occult blood positive
	Microscopic	Increased bacterial flora, starch (+), RBCs 2-5/high power field (40×)	Mild inflammatory findings
	Parasitology	*Giardia duodenalis *cysts (++)	Positive for intestinal parasites
Blood cultures	Peripheral culture #1	Negative	No growth
	Peripheral culture #2	Negative	No growth
Hepatic abscess drainage	Gram stain	6-10 leukocytes/field (100×), no bacteria	Inflammatory, no organisms
	Culture	Negative	No growth

The patient received analgesia, respiratory therapy, and antithrombotic prophylaxis. The clinical course was favorable, with progressive removal of drains and no additional complications, accompanied by a significant decrease in inflammatory markers and progressive improvement in liver function tests. He was discharged in good general condition, with recommendations for follow-up imaging, postoperative care, and outpatient follow-up with the thoracic surgery service.

Written informed consent was obtained from the patient for publication of this case and the accompanying images. 

## Discussion

HPF is an uncommon but clinically relevant complication of liver abscesses. It was first described in 1850 by Peacock in a patient with a hepatic hydatid cyst complicated by hydatid vomica [9]. Since then, although hepatic hydatidosis has historically been the most frequently reported cause, the epidemiology has evolved over recent decades. Pyogenic liver abscesses [10] and ALAs [11], as well as iatrogenic complications related to thoracic drainage placement, hepatic resection, or radiofrequency ablation, have emerged as relevant etiologies. Additional causes include biliary obstruction secondary to tumors, predominantly of the biliary tree, traumatic liver injuries, both blunt and penetrating, with or without expansile hematoma formation, and congenital conditions [4,5].

The pathophysiology involves diaphragmatic erosion secondary to local inflammatory processes and the pressure exerted by a subphrenic abscess, facilitating communication between the hepatic cavity and the pulmonary parenchyma. An alternative mechanism is direct invasion of the pulmonary parenchyma from an adjacent hepatic focus, with subsequent development of bronchorrhea [4,6].

Clinical recognition of this entity is often challenging, as its initial presentation may lack specific features and mimic primary respiratory infections, as occurred in the present case, in which pneumonia was initially suspected. Pure HPFs typically present with fever, leukocytosis, right upper quadrant pain, pleuritic pain, purulent cough, and variable degrees of dyspnea [3,4]. A key distinguishing feature is the nature of sputum: purulent bronchorrhea predominates in HPFs, whereas bilioptysis is pathognomonic of bronchobiliary fistulas [7,10]. In our case, the presence of brownish sputum may reflect the expectoration of necrotic material from the hepatic abscess through the transdiaphragmatic communication. 

From a diagnostic perspective, CT is the imaging modality of choice as it allows simultaneous evaluation of the liver, diaphragm, and lungs. According to Kontoravdis et al., characteristic findings include a subphrenic hepatic abscess adjacent to the diaphragm, right pleural effusion, basal atelectasis, and a pulmonary abscess in the right lower lobe [4]. Although pneumobilia has also been described, it is more typically associated with bronchobiliary fistula than with HPF and should therefore be interpreted in that context. 

In selected cases, complementary studies such as endoscopic retrograde cholangiopancreatography (ERCP), percutaneous transhepatic cholangiography (PTC), or magnetic resonance cholangiography (MRC) are recommended. These techniques allow confirmation of the fistulous tract and assessment of distal biliary obstruction; ERCP and PTC are both diagnostic and therapeutic [12]. 

Management of HPF remains clinically challenging and is based on three fundamental principles: control of the infectious focus, reduction of pressure within the fistulous tract, and treatment of the underlying pathology that favored its development [2,10]. In the context of ALA, initial management includes antiparasitic therapy and broad-spectrum antibiotics with gram-negative coverage, combined with CT-guided percutaneous drainage of hepatic or pulmonary abscesses to achieve adequate sepsis control [4,13].

Three main therapeutic strategies have been described: conservative management, minimally invasive techniques, and open surgery. Conservative approaches aim to promote spontaneous fistula closure through effective drainage of the thoracic cavity and biliary system, either endoscopically or percutaneously [4,14]. This strategy has proven effective in a subset of patients, particularly those with post-traumatic fistulas, with reported success rates of up to 60% [15]. However, prolonged drainage periods - often exceeding five weeks - may be required, and outcomes depend on the degree of residual inflammation and adequate infection control [4].

Historically, open surgery has been considered the gold standard as it allows definitive closure of the fistulous tract, with reported mortality rates of approximately 10.3% [4]. Nevertheless, contemporary clinical practice has shifted toward hybrid strategies combining surgical intervention with minimally invasive techniques, including endoscopic and interventional radiology procedures, thereby reducing the morbidity and mortality associated with major thoracoabdominal surgery. This comprehensive approach is based on the classical principles described by Ferguson and Burford, which include thoracotomy, adequate drainage of the hepatic bed, secure closure of the diaphragmatic defect using non-absorbable sutures, pulmonary decortication, pulmonary resection in cases of irreversible damage, and, in selected cases, prophylactic decompression of the biliary tree [2,4,16].

Currently, aggressive surgical management is reserved for chronic or complex fistulas, particularly those associated with progressive clinical deterioration, significant respiratory compromise, or uncontrolled thoracic or abdominal sepsis. Adjunctive alternative strategies have been reported, including bronchoscopic sealing of the fistulous tract [4] and the use of biological sealants or embolic materials, such as fibrin glue [17], cyanoacrylates [18], and histoacryl [19]; however, the available evidence supporting these techniques remains limited.

## Conclusions

HPF secondary to an ALA is an exceptionally rare and highly complex complication, associated with significant morbidity and mortality when diagnosis is delayed. A high index of suspicion is required, particularly in patients from endemic regions who present with liver abscesses and atypical respiratory symptoms, especially when imaging demonstrates a subdiaphragmatic hepatic abscess in close continuity with a basal pulmonary lesion and associated diaphragmatic disruption. Contrast-enhanced CT remains the diagnostic cornerstone. Management should be individualized according to disease severity and anatomical complexity; in advanced cases, particularly when percutaneous drainage is not feasible, surgery remains the standard for definitive source control.
